# The Voluntary Notification of Summer Diarrhœa

**Published:** 1909-05-08

**Authors:** 


### Diseases of Children.

### THE VOLUNTARY NOTIFICATION OF SUMMER DIARRHOEA.

The report of the results of the voluntary notification of zymotic enteritis in the Borough of Woolwich, by Dr. Sidney Davies, the Medical Officer of Health, is of some interest. In spite of the fact that it is based on only 844 notified cases, and that the system has been in force for only four years and during the months of July, August, and September, it confirms the views generally held on the causes of this fatal disease in infancy. Rather more than half the cases were under one year of age, rather more than one-fourth were in their second year, about 10 per cent. between two and five years, and 10 per cent. over this age. The mortality was 14 per cent., varying from 11 to 17 per cent. in the different years. As usual, the highest mortality was in the first three months of life, and the incidence of the disease greatest in the second three months. The mortality rate decreased as the age of the sufferers increased, and was least when the affection was least prevalent. No doubt diminished prevalence indicates diminished virulence and infectivity. Inquiry into the mode of feeding confirmed our knowledge of the value of breast-feeding as a preventive, and of the frequency of the disease in proportion to the frequency of erroneous methods of feeding. Depot milk proved valuable as a preventive. Not much importance can be attached to this, for, in addition to the cleanliness of the milk, the mothers are more or less supervised and advised on the methods of feeding and general cleanliness. Contrary to what is generally accepted, it does not appear that condensed milk is any less dangerous than fresh milk. The dietetic facts favour the opinion that all erroneous methods of feeding, which are liable to disturb the digestion and interfere with the nutrition and general well-being of the child, are predisposing causes of infection. The incubation period varied from two to seven days.

### The Causes of Epidemic Enteritis.

Overcrowding held its own as one of the factors affecting the prevalence of the disease, but, curiously, the evidence in favour of dirty houses as a cause received no support. The distribution of infected houses was more or less in groups, and suggested that the mode of spread in many instances was from the backs of the infected houses to the backs of those in the immediate neighbourhood, and the adjacent ones on each side. This mode of spread strongly supports the opinion commonly held that the disease is spread by flies carrying the infection from fæcal or infected material. Manure pits did not seem to affect the distribution, probably because the stables in Woolwich are not often situated among working-class dwellings. Privy middens are much more likely sources of infection. Manure pits may be breeding-grounds for flies, but, so far, there is no evidence that the specific organism of zymotic enteritis is there developed. Observations on the temperature of the air and the soil showed that a high reading of the 3-feet ground thermometer once reached is more generally coincident with epidemicity and virulence than the maintenance of a high average temperature throughout the quarter. Once a high-ground temperature is reached the infective agent multiplies freely outside the body and the disease becomes prevalent, even though the temperature falls. The maximum incidence was on an average three weeks later than the highest ground temperature. Rainfall and bright sunshine seemed to be merely subsidiary agents. A high ground temperature was a more important cause than a high atmospheric temperature.

Dr. Davies considers that the results of notification have been satisfactory, and that the system should be continued.

His main argument in support of this is based on a comparison of the death-rate in Woolwich and London in the summer quarters of the last eight years, but we fear that an analysis of his figures weakens their value very considerably. According to his table the death-date from diarrhœa was slightly higher in Woolwich than in London during the four years 1901-4, when voluntary notification was not in force at Woolwich; but much lower during the past four years—namely 1.37, as compared with 1.77 (given as 1.78 in the table). Analysis of the table, however, shows that in 1901 the death-rates in the two places were practically identical; in 1902 and 1903 the death-rate in Woolwich was only two-thirds of that in London; and that it is only the inclusion of the year 1904, in which the rate was 4.52 as compared with 3.39 in London, that enables him to obtain a mean average death-rate for Woolwich slightly higher than that for London. Nevertheless during the next four years the death-rate from diarrhœa was *each* year less in Woolwich than in the remainder of London.

It is almost a pity that the statistical argument is made use of at all. There is quite sufficient evidence in favour of notification without it. Its main value is preventive, for it leads to the investigation of each outbreak, and the adoption of measures for its prevention; the encouragement of and education in cleanliness and proper feeding; and the strict attention of the public health authorities to sanitary matters. It is more than probable that the abolition of middens and open privies, if it were possible, would lead to a great diminution in zymotic enteritis. Though we must realise the importance of these as breeding grounds of the specific organisms and of the flies which convey the infection to milk, food, and directly to the lips and nasal mucosa of the infant, yet there are other sources of infection, notably the diapers of a baby suffering from the disease. The occurrence of outbreaks in hospitals for children, in wards for babies, may be due to an infected milk supply, but is sometimes the result of carelessness in disposing of the infected articles of clothing, or neglect of washing the hands before preparing the food for the infant.

